# 1-[(Methyl­sulfon­yl)­oxy]pyridin-1-ium methane­sulfonate

**DOI:** 10.1107/S2414314621010269

**Published:** 2021-10-19

**Authors:** Tobias Taeufer, Anke Spannenberg, Jola Pospech

**Affiliations:** a Leibniz-Institut für Katalyse e. V., Albert-Einstein-Str. 29a, 18059 Rostock, Germany; University of Aberdeen, Scotland

**Keywords:** crystal structure, pyridinium cation, sulfonate anion

## Abstract

The title mol­ecular salt, C_6_H_8_NO_3_S^+^·CH_3_O_3_S^−^, consists of a cationic sulfonated pyridine *N*-oxide moiety and a methane­sulfonate anion. An N—O bond length of 1.4004 (15) Å is observed in the cation. In the crystal, weak C—H⋯O inter­actions link the components into a three-dimensional network.

## Abstract 2

The title compound is a pyridinium salt that consists of a sulfonated pyridine *N*-oxide cation and a methane­sulfonate anion.

## Graphical abstract

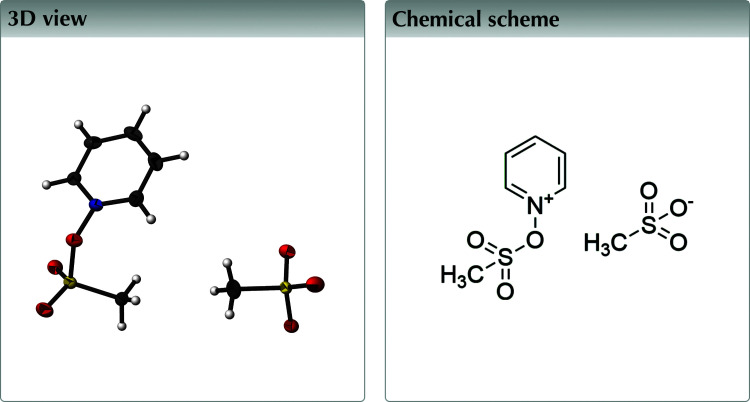

## Structure description

Zhen-Chu & Stang (1984 ▸) reported the synthesis of 1-{[(tri­fluoro­meth­yl)sulfon­yl]­oxy}pyridin-1-ium tri­fluoro­methane­sulfonate from pyridine *N*-oxide and tri­fluoro­methane­sulfonic anhydride. The reactivity of *O*-sulfonyl pyridinium salts toward nucleophiles and their substitution of the 2-position as reaction products were described by Umemoto *et al.* (1996 ▸). Rössler *et al.* (2019 ▸) reported the photochemical application of 1-{[(tri­fluoro­meth­yl)sulfon­yl]­oxy}pyridin-1-ium tri­fluoro­methane­sulfonate, which allows direct amination of arenes and heteroarenes.

**Figure 1:**
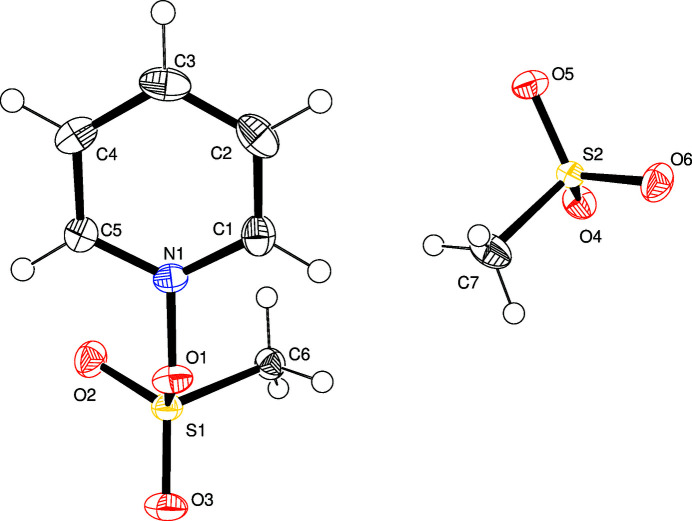
Mol­ecular structure of the title compound with displacement ellipsoids drawn at the 50% probability level.

Here, we report the formation of 1-[(methyl­sulfon­yl)­oxy]pyridin-1-ium methane­sulfonate, C_6_H_8_NO_3_S^+^·CH_3_O_3_S^−^, obtained from the reaction of pyridine-*N*-oxide and methane­sulfonic anhydride. Its mol­ecular structure (Fig. 1 ▸) consists of a cationic sulfonated pyridine *N*-oxide moiety and a methane­sulfonate anion. The N—O bond length of 1.4004 (15) Å is similar to that observed in 1-{[(tri­fluoro­meth­yl)sulfon­yl]­oxy}pyridin-1-ium tri­fluoro­methane­sulfonate [N—O = 1.4095 (11) Å; Rössler *et al.*, 2019 ▸]. Furthermore, O1 is 0.19 Å out of the pyridinium plane in the title compound and the N1—O1—S1—C6 torsion angle is 66.72 (11)°.

In the crystal, the components are linked by C—H⋯O inter­actions into a three-dimensional network (Table 1 ▸); the C5—H5⋯O4 bond with H⋯O = 2.19 Å is notably short.

## Synthesis and crystallization

Following a modified literature procedure of Rössler *et al.* (2019 ▸), a stirred solution of pyridine *N*-oxide (3.00 g, 31.6 mmol, 1.0 eq.) in DCM (100 ml) was treated dropwise with a solution of methane­sulfonic anhydride (7.13 g, 37.9 mmol, 1.3 eq.) in DCM at −30°C. After complete addition, the reaction mixture was stirred for 2 h and allowed to warm to room temperature. The white precipitate was filtered and washed with fresh DCM (30 ml). Additional drying *in vacuo* yields the title compound (6.40 g, 23.8 mmol, 75%). Colourless needles suitable for X-ray crystal structure analysis were obtained by cooling a warm saturated aceto­nitrile solution to −30°C (Caution: heating to > 50°C leads to decomposition of the title compound.). ^1^H NMR (400 MHz, aceto­nitrile-*d*_3_) *δ* 8.7 (*s*, 2H), 8.1 (*s*, 1H), 7.9 (*s*, 2H), 3.5 (*s*, 2H), 2.6 (*s*, 3H). ^13^C NMR (101 MHz, aceto­nitrile-*d*_3_) *δ* 140.62, 129.10, 41.78, 39.65. IR (ATR, neat, cm^−1^): 3108 (*w*), 3013 (*w*), 2986 (*w*), 2943 (*w*), 1606 (*w*), 1479 (*w*), 1428 (*w*), 1381 (*m*), 1330 (*w*), 1315 (*w*), 1289 (*w*), 1182 (*s*), 1163 (*s*), 1144 (*m*), 1040 (*s*), 1002 (*m*), 984 (*s*), 818 (*m*), 789 (*s*), 762 (*s*), 672 (*m*), 655 (*s*), 602 (*w*), 554 (*s*), 520 (*s*), 507 (*s*), 489 (*m*), 456 (*m*), 421 (*m*). Analysis (%) calculated for C_7_H_11_NO_6_S_2_: C, 31.22; H, 4.12; N, 5.20; S, 23.81. Found: C, 31.02; H, 4.61; N, 4.93; S, 23.62.

## Refinement

Crystal data, data collection and structure refinement details are summarized in Table 2 ▸.

## Supplementary Material

Crystal structure: contains datablock(s) I. DOI: 10.1107/S2414314621010269/hb4392sup1.cif

Structure factors: contains datablock(s) I. DOI: 10.1107/S2414314621010269/hb4392Isup2.hkl

Supporting information file. DOI: 10.1107/S2414314621010269/hb4392Isup3.cml

CCDC reference: 2113673

Additional supporting information:  crystallographic information; 3D view; checkCIF report

## Figures and Tables

**Table 1:** Hydrogen-bond geometry (Å, °)

*D*—H⋯*A*	*D*—H	H⋯*A*	*D*⋯*A*	*D*—H⋯*A*
C1—H1⋯O5^i^	0.95	2.29	3.1745 (19)	154
C3—H3⋯O4^ii^	0.95	2.35	3.132 (2)	139
C4—H4⋯O2^iii^	0.95	2.42	3.254 (2)	146
C5—H5⋯O4^iv^	0.95	2.19	3.1008 (19)	159
C6—H6*A*⋯O5^v^	0.98	2.39	3.1840 (19)	137
C6—H6*B*⋯O5^i^	0.98	2.38	3.3023 (19)	157
C6—H6*C*⋯O6^vi^	0.98	2.36	3.261 (2)	152

Symmetry codes: (i) 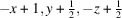 ; (ii) 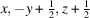 ; (iii) 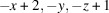 ; (iv) 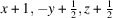 ; (v) 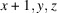 ; (vi) 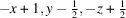 .

**Table 2:** Experimental details

Crystal data	
Chemical formula	C_6_H_8_NO_3_S^+^·CH_3_O_3_S^−^
*M*_r_	269.29
Crystal system, space group	Monoclinic, *P*2_1_/*c*
Temperature (K)	150
*a*, *b*, *c* (Å)	7.9335 (3), 7.6255 (3), 18.3875 (7)
β (°)	99.0734 (14)
*V* (Å^3^)	1098.47 (7)
*Z*	4
Radiation type	Mo *K*α
μ (mm^−1^)	0.50
Crystal size (mm)	0.36 × 0.08 × 0.08

Data collection	
Diffractometer	Bruker APEXII CCD
Absorption correction	Multi-scan (*SADABS*; Bruker, 2014 ▸)
*T*_min_, *T*_max_	0.84, 0.96
No. of measured, independent and observed [*I* > 2σ(*I*)] reflections	27689, 3400, 2732
*R*_int_	0.033
(sin θ/λ)_max_ (Å^−1^)	0.718

Refinement	
*R*[*F* ^2^ > 2σ(*F* ^2^)], *wR*(*F* ^2^), *S*	0.035, 0.098, 1.05
No. of reflections	3400
No. of parameters	147
H-atom treatment	H-atom parameters constrained
Δρ_max_, Δρ_min_ (e Å^−3^)	0.48, −0.29

Computer programs: *APEX2* (Bruker, 2014 ▸), *SAINT* (Bruker, 2013 ▸), *SHELXS97* (Sheldrick, 2008 ▸), *SHELXL2018/3* (Sheldrick, 2015 ▸), *ORTEP-3 for Windows* (Farrugia, 2012 ▸) and *publCIF* (Westrip, 2010 ▸).

## full crystallographic data

### Crystal data

**Table:**

C_6_H_8_NO_3_S^+^·CH_3_O_3_S^−^	*F*(000) = 560
*M**_r_* = 269.29	*D*_x_ = 1.628 Mg m^−^^3^
Monoclinic, *P*2_1_/*c*	Mo *K*α radiation, λ = 0.71073 Å
*a* = 7.9335 (3) Å	Cell parameters from 8765 reflections
*b* = 7.6255 (3) Å	θ = 2.2–30.6°
*c* = 18.3875 (7) Å	µ = 0.50 mm^−^^1^
β = 99.0734 (14)°	*T* = 150 K
*V* = 1098.47 (7) Å^3^	Needle, colourless
*Z* = 4	0.36 × 0.08 × 0.08 mm

### Data collection

**Table:**

Bruker APEXII CCD diffractometer	3400 independent reflections
Radiation source: fine-focus sealed tube	2732 reflections with *I* > 2σ(*I*)
Detector resolution: 8.3333 pixels mm^-1^	*R*_int_ = 0.033
φ and ω scans	θ_max_ = 30.7°, θ_min_ = 2.2°
Absorption correction: multi-scan (SADABS; Bruker, 2014)	*h* = −11→11
*T*_min_ = 0.84, *T*_max_ = 0.96	*k* = −10→10
27689 measured reflections	*l* = −26→26

### Refinement

**Table:**

Refinement on *F*^2^	Primary atom site location: structure-invariant direct methods
Least-squares matrix: full	Hydrogen site location: inferred from neighbouring sites
*R*[*F*^2^ > 2σ(*F*^2^)] = 0.035	H-atom parameters constrained
*wR*(*F*^2^) = 0.098	*w* = 1/[σ^2^(*F*_o_^2^) + (0.0491*P*)^2^ + 0.5052*P*] where *P* = (*F*_o_^2^ + 2*F*_c_^2^)/3
*S* = 1.05	(Δ/σ)_max_ = 0.001
3400 reflections	Δρ_max_ = 0.48 e Å^−^^3^
147 parameters	Δρ_min_ = −0.29 e Å^−^^3^
0 restraints	

### Special details

**Table:**

Geometry. All esds (except the esd in the dihedral angle between two l.s. planes) are estimated using the full covariance matrix. The cell esds are taken into account individually in the estimation of esds in distances, angles and torsion angles; correlations between esds in cell parameters are only used when they are defined by crystal symmetry. An approximate (isotropic) treatment of cell esds is used for estimating esds involving l.s. planes.

### Fractional atomic coordinates and isotropic or equivalent isotropic displacement parameters (Å^2^)

**Table:**

	*x*	*y*	*z*	*U*_iso_*/*U*_eq_	
C1	0.7438 (2)	0.3898 (2)	0.41962 (9)	0.0246 (3)	
H1	0.710677	0.472427	0.381358	0.030*	
C2	0.6250 (2)	0.3078 (3)	0.45464 (10)	0.0314 (4)	
H2	0.507103	0.332900	0.440638	0.038*	
C3	0.6775 (2)	0.1881 (2)	0.51058 (10)	0.0297 (4)	
H3	0.595713	0.131149	0.534962	0.036*	
C4	0.8495 (2)	0.1523 (2)	0.53060 (9)	0.0248 (3)	
H4	0.886377	0.070444	0.568762	0.030*	
C5	0.96647 (19)	0.2354 (2)	0.49509 (8)	0.0203 (3)	
H5	1.085198	0.213089	0.508170	0.024*	
C6	0.95043 (19)	0.3470 (2)	0.27306 (8)	0.0215 (3)	
H6A	0.993775	0.324167	0.226919	0.032*	
H6B	0.886302	0.457327	0.268887	0.032*	
H6C	0.875225	0.250804	0.282868	0.032*	
C7	0.5041 (2)	0.4202 (3)	0.19949 (11)	0.0349 (4)	
H7A	0.549811	0.518471	0.174279	0.052*	
H7B	0.583596	0.321159	0.202500	0.052*	
H7C	0.489657	0.456406	0.249278	0.052*	
N1	0.90840 (16)	0.34870 (16)	0.44154 (7)	0.0182 (2)	
O1	1.02878 (14)	0.45125 (15)	0.41284 (6)	0.0228 (2)	
O2	1.18327 (14)	0.19494 (16)	0.36976 (7)	0.0269 (3)	
O3	1.23496 (15)	0.50071 (17)	0.33542 (7)	0.0295 (3)	
O4	0.33727 (14)	0.30518 (17)	0.07686 (6)	0.0259 (3)	
O5	0.24872 (14)	0.20919 (15)	0.19056 (6)	0.0252 (2)	
O6	0.19265 (15)	0.50693 (16)	0.14815 (7)	0.0285 (3)	
S1	1.12073 (5)	0.36235 (5)	0.34493 (2)	0.01954 (10)	
S2	0.30549 (4)	0.35686 (5)	0.15000 (2)	0.01779 (10)	

### Atomic displacement parameters (Å^2^)

**Table:**

	*U*^11^	*U*^22^	*U*^33^	*U*^12^	*U*^13^	*U*^23^
C1	0.0256 (7)	0.0232 (8)	0.0244 (8)	0.0051 (6)	0.0019 (6)	−0.0025 (6)
C2	0.0202 (7)	0.0375 (10)	0.0370 (9)	0.0025 (7)	0.0063 (7)	−0.0092 (8)
C3	0.0301 (8)	0.0286 (9)	0.0345 (9)	−0.0074 (7)	0.0174 (7)	−0.0081 (7)
C4	0.0364 (9)	0.0188 (7)	0.0211 (7)	−0.0004 (6)	0.0103 (6)	−0.0008 (6)
C5	0.0234 (7)	0.0191 (7)	0.0185 (7)	0.0017 (6)	0.0037 (5)	−0.0021 (5)
C6	0.0213 (7)	0.0232 (8)	0.0196 (7)	−0.0004 (6)	0.0018 (5)	−0.0006 (6)
C7	0.0247 (8)	0.0409 (11)	0.0374 (10)	−0.0099 (8)	−0.0006 (7)	−0.0060 (8)
N1	0.0205 (6)	0.0158 (6)	0.0191 (6)	−0.0019 (5)	0.0058 (5)	−0.0019 (5)
O1	0.0279 (5)	0.0188 (5)	0.0228 (5)	−0.0065 (4)	0.0080 (4)	−0.0023 (4)
O2	0.0254 (6)	0.0273 (6)	0.0288 (6)	0.0067 (5)	0.0068 (5)	0.0053 (5)
O3	0.0290 (6)	0.0309 (7)	0.0301 (6)	−0.0125 (5)	0.0092 (5)	0.0003 (5)
O4	0.0234 (5)	0.0336 (7)	0.0218 (5)	0.0011 (5)	0.0066 (4)	−0.0027 (5)
O5	0.0286 (6)	0.0198 (6)	0.0284 (6)	−0.0039 (5)	0.0082 (5)	0.0022 (5)
O6	0.0326 (6)	0.0242 (6)	0.0304 (6)	0.0097 (5)	0.0098 (5)	0.0047 (5)
S1	0.01875 (17)	0.01993 (19)	0.02037 (18)	−0.00212 (13)	0.00443 (13)	0.00094 (13)
S2	0.01614 (16)	0.01827 (18)	0.01905 (18)	−0.00070 (12)	0.00307 (12)	0.00026 (13)

### Geometric parameters (Å, º)

**Table:**

C1—N1	1.3418 (19)	C6—H6B	0.9800
C1—C2	1.373 (2)	C6—H6C	0.9800
C1—H1	0.9500	C7—S2	1.7586 (17)
C2—C3	1.389 (3)	C7—H7A	0.9800
C2—H2	0.9500	C7—H7B	0.9800
C3—C4	1.383 (3)	C7—H7C	0.9800
C3—H3	0.9500	N1—O1	1.4004 (15)
C4—C5	1.371 (2)	O1—S1	1.6847 (11)
C4—H4	0.9500	O2—S1	1.4188 (12)
C5—N1	1.336 (2)	O3—S1	1.4195 (12)
C5—H5	0.9500	O4—S2	1.4610 (12)
C6—S1	1.7384 (15)	O5—S2	1.4605 (12)
C6—H6A	0.9800	O6—S2	1.4500 (12)
			
N1—C1—C2	117.39 (15)	S2—C7—H7B	109.5
N1—C1—H1	121.3	H7A—C7—H7B	109.5
C2—C1—H1	121.3	S2—C7—H7C	109.5
C1—C2—C3	119.94 (15)	H7A—C7—H7C	109.5
C1—C2—H2	120.0	H7B—C7—H7C	109.5
C3—C2—H2	120.0	C5—N1—C1	125.35 (14)
C4—C3—C2	119.65 (16)	C5—N1—O1	117.54 (12)
C4—C3—H3	120.2	C1—N1—O1	116.47 (13)
C2—C3—H3	120.2	N1—O1—S1	117.09 (9)
C5—C4—C3	119.68 (16)	O2—S1—O3	120.71 (7)
C5—C4—H4	120.2	O2—S1—O1	107.09 (7)
C3—C4—H4	120.2	O3—S1—O1	98.72 (7)
N1—C5—C4	117.98 (14)	O2—S1—C6	111.99 (8)
N1—C5—H5	121.0	O3—S1—C6	113.03 (8)
C4—C5—H5	121.0	O1—S1—C6	102.42 (7)
S1—C6—H6A	109.5	O6—S2—O5	112.42 (7)
S1—C6—H6B	109.5	O6—S2—O4	112.76 (7)
H6A—C6—H6B	109.5	O5—S2—O4	111.97 (7)
S1—C6—H6C	109.5	O6—S2—C7	107.14 (9)
H6A—C6—H6C	109.5	O5—S2—C7	105.72 (9)
H6B—C6—H6C	109.5	O4—S2—C7	106.25 (8)
S2—C7—H7A	109.5		
			
O1—N1—C1—C2	−171.14 (14)	C2—C1—N1—C5	−0.5 (2)
N1—C1—C2—C3	0.2 (3)	C2—C1—N1—O1	−171.14 (14)
C1—C2—C3—C4	0.0 (3)	C5—N1—O1—S1	85.36 (14)
C2—C3—C4—C5	0.1 (3)	C1—N1—O1—S1	−103.28 (13)
C3—C4—C5—N1	−0.3 (2)	N1—O1—S1—O2	−51.23 (11)
C4—C5—N1—C1	0.6 (2)	N1—O1—S1—O3	−177.23 (10)
C4—C5—N1—O1	171.09 (13)	N1—O1—S1—C6	66.72 (11)

### Hydrogen-bond geometry (Å, º)

**Table:**

*D*—H···*A*	*D*—H	H···*A*	*D*···*A*	*D*—H···*A*
C1—H1···O5^i^	0.95	2.29	3.1745 (19)	154
C3—H3···O4^ii^	0.95	2.35	3.132 (2)	139
C4—H4···O2^iii^	0.95	2.42	3.254 (2)	146
C5—H5···O4^iv^	0.95	2.19	3.1008 (19)	159
C6—H6*A*···O5^v^	0.98	2.39	3.1840 (19)	137
C6—H6*B*···O5^i^	0.98	2.38	3.3023 (19)	157
C6—H6*C*···O6^vi^	0.98	2.36	3.261 (2)	152

Symmetry codes: (i) −*x*+1, *y*+1/2, −*z*+1/2; (ii) *x*, −*y*+1/2, *z*+1/2; (iii) −*x*+2, −*y*, −*z*+1; (iv) *x*+1, −*y*+1/2, *z*+1/2; (v) *x*+1, *y*, *z*; (vi) −*x*+1, *y*−1/2, −*z*+1/2.
